# Metabolic predictors of impaired glucose tolerance and type 2 diabetes in a predisposed population – A prospective cohort study

**DOI:** 10.1186/s12902-015-0048-8

**Published:** 2015-09-25

**Authors:** Josefin Henninger, Ann Hammarstedt, Araz Rawshani, Björn Eliasson

**Affiliations:** The Lundberg Laboratory for Diabetes Research, Department of Molecular and Clinical Medicine, the Sahlgrenska Academy at the University of Gothenburg, Gothenburg, Sweden; The Lundberg Laboratory for Diabetes Research, Sahlgrenska University Hospital, 413 45 Gothenburg, Sweden

## Abstract

**Background:**

We characterized in detail (oral and intravenous glucose tolerance tests (OGTT and IVGTT), euglycemic hyperinsulinemic clamp, adipose tissue biopsy), healthy first-degree relatives (FDR) of individuals with type 2 diabetes (T2D), to examine predictive factors for future development of impaired glucose tolerance (IGT) or T2D.

**Methods:**

Non-diabetic FDR (*n* = 138, mean age 40.5 ± 6.5 years, 57 % women) underwent an extended OGTT every 3 years to assess any deterioration in glucose tolerance status. Differences between groups were assessed by logistic fit for continuous variables and by contingency analysis for categorical variables. Multiple logistic regression analysis was applied to adjust for confounding variables.

**Results:**

At follow-up (mean 5.6 ± 2.4 years) 19 subjects had IGT and 4 had T2D. At baseline these 23 subjects had more family members with T2D, higher fasting plasma glucose, higher OGTT plasma glucose at 120 min, higher HbA1c, lower M-value and higher total cholesterol compared to subjects with normal glucose tolerance (NGT). There were significantly larger changes in weight, BMI, fasting plasma glucose, OGTT plasma glucose at 120 min and HbA1c in individuals developing IGT or T2D during the follow-up period than the subjects remaining NGT.

Crude predictors of deteriorating glucose tolerance were age, family history of diabetes and of hypertension, OGTT plasma glucose levels at 60 min, 90 min, and 120 min, as well as serum bilirubin, ALP and creatinine (*p-*values <0.05). A multiple nominal logistic regression model revealed that male sex, low M-value and high physical exercise (*p-*values <0.05) predicted development of IGT/T2DM.

**Conclusion:**

In sum, genetically predisposed individuals for T2D with deteriorating glucose tolerance exhibit insulin resistance as well as beta-cell and signs of adipose tissue dysfunction, emphasizing the multifactorial pathophysiology in the development of IGT and T2D.

## Background

The rapid global increase in the number of individuals with type 2 diabetes (T2D) was considered caused by increasing life expectancy, sedentary lifestyle and disadvantageous diet habits, possibly in combination with genetic and environmental factors [1, 2]. In spite of only weak associations between specific genes and the development of T2D, first-degree relatives (FDR) of T2D patients are clearly at an elevated risk of developing the same disease, proportionally with the number of family members affected [1, 3, 4].

The pathophysiology of T2D has been extensively studied, and the current view is that it is indeed multifactorial [5]. The basis is a combination of impairments in insulin secretion and sensitivity, as well as increased hepatic glucose production, although disturbances in glucagon production, incretin effects, renal function and appetite regulation also may play important roles. Adipocyte dysfunction and its associated adipocyte cell hypertrophy and production of pro-inflammatory cytokines, such as interleukin 6 (IL-6) and tumor necrosis factor alpha (TNF-α), also contribute to the development of insulin resistance and T2D [5].

In FDR, genetic and intrauterine factors, as well as lifestyle-related acquired factors associated with insulin resistance and pancreatic beta cell dysfunction, have been proposed to constitute the increased susceptibility to T2D [3, 6–11]. Boesgaard et al. have shown that there is indeed an association between specific genes and both beta cell dysfunction and insulin resistance in this group of individuals [8, 9]. Studies have also demonstrated that normoglycaemic FDR exhibit dyslipidemia, including postprandial hypertriglyceridemia which is closely linked to glucose intolerance, and anthropometric risk factors such as higher body mass index (BMI) and waist circumference compared with individuals without family history of T2D [11–13]. We have also recently demonstrated that adipocyte and adipose tissue dysfunction with remodeling and fibrosis are likely to contribute to the increased risk of developing T2D in FDR [14, 15].

In this prospective cohort study of 138 non-diabetic FDR we set out to explore predictive factors in glucose metabolism and adipocyte function for future development of IGT and T2D. We used intravenous glucose tolerance tests, euglycemic hyperinsulinemic clamps and subcutaneous adipose tissue biopsies to characterize in detail insulin secretion and sensitivity, as well as adipocyte function, and to study correlations between these.

## Methods

### Ethics statement

The study protocol was approved by the local Ethical Committees at the Sahlgrenska Academy at the University of Gothenburg (S 655–03) and was performed in agreement with the Declaration of Helsinki. All subjects received oral and written information and gave written consent to participate.

### Participants

We recruited 138 FDR (mean age 40.5 ± 6.5 years, 57 % women) via newspaper advertisements. We used the following inclusion criteria: at least one first-degree relative with T2D, age 18–40 years, general good health, and no continuous medication. T2D, established by fasting plasma glucose values and an oral glucose tolerance test (OGTT), was an exclusion criterion. Specifically, no continuous medication was a required criterion also at follow-up examinations. We used the World Health Organization criteria for impaired glucose tolerance and diabetes mellitus [16].

### Measurements at baseline

Anthropometric data was collected at the first visit. Body weight and height, and waist and hip circumferences were recorded, BMI was calculated, and the proportions of body fat and lean body mass (LBM) were determined using bioelectrical impedance (single frequency, 50 kHz; Animeter, HTS, Odense, Denmark). Blood pressure was measured in a sitting position after a five minutes rest with a mercury sphygmomanometer. High physical activity was defined as exercising four or more times a week and accentuated family history of T2D (FH+) was defined as having more than one relative with the disease, including one FDR.

A subcutaneous abdominal adipose tissue biopsy was performed. The biopsies (approximately 20–30 mg) were obtained with a needle aspiration technique, from the paraumbilical region after local infiltrative anesthesia with lidocaine (20 ml, 0.5 %).

On a separate occasion, fasting blood samples were drawn after 12 h of fasting and were followed by an OGTT (75 g glucose) to evaluate glucose tolerance. Samples for measurement of plasma glucose and serum insulin were drawn after 0, 30, 90 and 120 min. Fasting plasma insulin and fasting plasma glucose were used to calculate a HOMA-IR index using the formula HOMA-IR = (fasting plasma glucose x fasting plasma insulin) / 22.5 [17].

On a third occasion, again after 12 h of fasting, an intravenous glucose tolerance test (IVGTT) was first performed to determine the first and second phases of insulin secretion. A bolus of glucose (300 mg/kg in a 50 % solution) was given within 30 s into an antecubital vein. Samples for the measurement of plasma glucose and insulin (arterialised venous blood) were drawn at −5, 0, 2, 4, 6, 8, 10, 20, 30, 40, 50 and 60 min. The acute and the late insulin responses, i.e. incremental area under the insulin curve, (AIR, 0–10 min; LIR, 10–60 min) were calculated using the trapezoidal method.

Thereafter, 60 min after the glucose bolus, a hyperinsulinemic euglycaemic clamp was initiated (insulin infusion: 240 pmol m-^2^ min-^1^ for 120 min) to evaluate insulin sensitivity [18]. Whole blood glucose was clamped at 5.0 mmol/l for the next 120 min by infusion of 20 % glucose at various rates according to glucose measurements performed at five minutes intervals (YSI, Yellow Springs Instrument Company, OH). Insulin sensitivity (M) was calculated as the mean glucose infusion rate during the last 30 min of the clamp adjusted for lean body mass, and M/I was calculated as the M-value corrected for steady-state insulin concentrations.

### Follow-up examinations

Anthropometric measurements, fasting blood samples, and an OGTT were performed every three years to assess any deterioration in glucose tolerance, defined as IGT and/or T2D at follow-up.

### Laboratory analyses

Fasting levels of plasma glucose, insulin, and blood lipids were measured using standard laboratory methods (Department of Chemistry, Sahlgrenska University Hospital, Gothenburg, Sweden). LDL cholesterol was calculated using the Friedewald equation [19]. HbA1c was determined using high-performance liquid chromatography (Mono-S method). In this study, all HbA1c values were converted to International Federation of Clinical Chemistry and Laboratory Medicine (IFCC) standard levels using the formula: HbA1c (IFCC) = (10.45 × HbA1c (Mono-S) -10.62 [20]. Plasma insulin was measured at the University of Tübingen, Germany, by micro-particle enzyme immunoassay (Abbott Laboratories, Tokyo, Japan).

Total circulating serum adiponectin concentrations were measured by an ultrasensitive ELISA (B-Bridge, Sunnyvale, CA). Plasma IL-6 levels were quantified using an IL-6 specific proliferation bioassay. IL-6 concentrations were calculated using dilutions of recombinant human IL-6 (Genzyme, Cambridge, Massachusetts, USA) as previously described [21].

### Adipocyte cell size measurement

Isolation of adipocytes after the biopsy was performed essentially as previously described [22]. Briefly, biopsies were washed to remove traces of blood and treated with collagenase (1 mg/ml) (Sigma, St Louise, MO, USA) for 60 min at 37 °C in a shaking water bath. Isolated adipocytes were filtered through a 250 mm nylon mesh and washed with fresh medium. Adipocyte cells were placed on a siliconized glass slide and 100 consecutive cell diameters were measured with a calibrated ocular and expressed as the average value in μm.

### Cell lysates and immunoblotting

Adipose tissue specimens obtained from needle biopsies were treated as described above. Cells were then lysed in the presence of protease inhibitors followed by protein separation by SDS-PAGE as described [23].

### Statistical analysis

The results for continuous variables are given as means ± one standard deviation and the results for categorical variables are given as frequencies. A value of p < 0.05 was considered statistically significant. All variables were visually assessed for normality and considered normally distributed. The differences between the groups were assessed by logistic fit for continuous variables and by contingency analysis and specifically Pearson Chi-square tests, for categorical variables. Multiple linear/logistic regression analyses were applied to adjust for confounding variables. Data analyses were performed using JMP version 10.0 and SAS 9.1.3 (SAS Institute Inc., Cary, North Carolina, USA), as well as R Project for Statistical Computing version 3.1.2. (Vienna, Austria: R Foundation for Statistical Computing).

## Results

### Baseline and follow-up results

Clinical and metabolic characteristics at baseline are given in Tables 1 and 2. There were 138 individuals at baseline, whereof 21 exhibited IGT. The IGT group had slightly but significantly higher BMI, as well as higher fasting plasma glucose and insulin levels, OGTT plasma glucose at 120 min, HOMA-IR, total and LDL cholesterol, serum triglycerides and adipocyte cell size, while insulin sensitivity (M-values and M/I) and HDL cholesterol were lower.

**Table 1:** Clinical characteristics of all subjects at baseline, also stratified by glucose tolerance status at baseline

	All	NGT	IGT	*P-*value (NGT-IGT)
*N*	138	117	21	
Age (years)	40.5 ± 6.5	40.2 ± 6.7	42.5 ± 5.0	n.s.
Sex (% male)	60 (43 %)	51 (44 %)	9 (43 %)	n.s.
Weight (kg)	77.1 ± 13.0	76.8 ± 12.8	79.5 ± 14.4	n.s.
BMI (kg/m^2^)	25.2 ± 3.4	25.0 ± 3.3	26.7 ± 3.7	0.049
Waist (cm)	88.9 ± 10.4	88.3 ± 10.1	92.6 ± 11.4	n.s.
Waist/hip circumference ratio	0.87 ± 0.1	0.86 ± 0.1	0.89 ± 0.1	n.s.
Fat percent (%)	25.8 ± 7.8	25.2 ± 8.0	28.8 ± 6.0	0.058
Systolic blood pressure (mmHg)	118 ± 11	118 ± 10	122 ± 14	0.098
Diastolic blood pressure (mmHg)	75 ± 9	75 ± 9	79 ± 10	0.081
Currently smoking	13 (10 %)	13 (12 %)	0 (0 %)	n.s.
High physical activity	46 (35 %)	43 (38 %)	3 (17 %)	0.074
High heredity for T2D	78 (59 %)	68 (61 %)	10 (48 %)	0.092

NGT = normal glucose tolerance. IGT = impaired glucose tolerance. Data are means ± standard deviation. *P-*values below 0.1 are given numerically, otherwise stated as not significant (n.s.)

**Table 2:** Metabolic characteristics of all subjects at baseline, also stratified by glucose tolerance status at baseline

	All	NGT	IGT	*P-*value (NGT-IGT)
*N*	138	117	21	
Fasting plasma glucose (mmol/L)	4.8 ± 0.4	4.8 ± 0.4	5.1 ± 0.6	0.006
Fasting plasma insulin (pmol/L)	49.3 ± 31.9	45.1 ± 27.0	69.1 ± 44.4	0.013
OGTT 2 h plasma glucose (mmol/L)	6.0 ± 1.6	5.5 ± 1.1	8.7 ± 0.7	<0.001
HbA1c (mmol/mol)	32.5 ± 2.4	32.6 ± 2.3	32.1 ± 3.3	n.s.
M-value (GIR/lbm/min)	12.8 ± 3.8	13.3 ± 3.7	10.21 ± 3.4	0.002
M/I (GIR/lbm/min/pmol/L)	0.022 ± 0.01	0.023 ± 0.01	0.017 + 0.01	0.010
IVGTT AIR (pmol/L x min)	3187 ± 2014	3291 ± 2092	2547 ± 1303	n.s.
IVGTT LIR (pmol/L x min)	7760 ± 4354	7493 ± 4177	9427 ± 5150	0.092
HOMA-IR (mmol x mU/L^2^)	10.6 ± 7.5	9.5 ± 6.1	15.8 ± 11.0	0.009
Serum cholesterol (mmol/L)	4.9 ± 0.9	4.8 ± 0.9	5.3 ± 0.8	0.016
Serum HDL (mmol/L)	1.6 ± 0.4	1.6 ± 0.4	1.4 ± 0.3	0.029
Serum triglycerides (mmol/L)	1.0 ± 0.5	1.0 ± 0.5	1.4 ± 0.7	0.012
Serum LDL (mmol/L)	2.9 ± 0.8	2.8 ± 0.7	3.3 ± 0.9	0.004
Urine albumin (>20 mg/L)	69 (51 %)	55 (51 %)	8 (44 %)	
Serum ALT (μkat/L)	0.43 ± 0.27	0.42 ± 0.24	0.50 ± 0.39	n.s.
Serum CRP (mg/L)	1.2 ± 2.0	1.1 ± 2.1	1.5 ± 1.4	n.s.
Serum creatinine (μmol/L)	88 ± 16	88 ± 16	87 ± 15	n.s.
Adipocyte cell size (μm)	94.3 ± 12.7	93.2 ± 13.1	101.1 ± 7.7	0.017
Serum adiponectin (μg/mL)	9.1 ± 4.6	9.3 ± 4.6	8.0 ± 4.3	n.s.
Il-6 (ng/mL)	45.3 ± 21.7	44.8 ± 21.4	49.3 ± 24.3	n.s.

NGT = normal glucose tolerance. IGT = impaired glucose tolerance. IL-6 = interleukin-6. Data are mean ± standard deviation. *P-*values have been calculated as stated in Methods. *P-*values below 0.1 are given numerically, otherwise stated as not significant (n.s.)

The mean follow-up time was 5.6 ± 2.4 years. At their last follow-up visit, 112 subjects were normoglycaemic, 19 had IGT and 4 had developed T2D. 3 individuals were lost to follow-up. Examining only individuals who were normoglycaemic at baseline (*n* = 114), clinical and metabolic characteristics at baseline are given in Table 3, grouped according to glucose tolerance status at follow-up. There were statistically significant differences between groups in follow-up time, age, family history of diabetes and OGTT plasma glucose levels at 120 min between subjects remaining NGT compared to the ones developing IGT/T2D. At the end of the follow-up period (5.3 and 6.9 years, respectively; *p =* 0.0028), there were significantly larger increases only in fat percentage and OGTT plasma glucose at 120 min in the individuals with IGT/T2D at the end of follow-up (data given in Table 4).

**Table 3:** Baseline characteristics of subjects with NGT at baseline, according to glycemic control status at follow-up

	NGT-NGT	NGT-IGT/T2D	*P-*value
*N*	100	14	
Follow-up time (years)	5.3 ± 2.4	6.9 ± 2.2	0.028
Age (years)	39.5 ± 6.8	43.6 ± 5.3	0.039
Sex (% male)	46 (46 %)	4 (28,5 %)	n.s.
Weight (kg)	77.2 ± 13.1	74.7 ± 11.6	n.s.
BMI (kg/m^2^)	24.9 ± 3.3	25.4 ± 3.3	n.s.
Waist (cm)	88.1 ± 10.3	89.1 ± 10.1	n.s.
Waist/hip circumference ratio	0.86 ± 0.09	0.87 ± 0.08	n.s.
Systolic blood pressure (mmHg)	117 ± 10	121 ± 9	n.s.
Diastolic blood pressure (mmHg)	75 ± 9	74 ± 6	n.s.
Fat percent (%)	24.7 ± 8.1	28.4 ± 7.3	n.s.
High heredity for T2D	56 (59 %)	11 (79 %)	<0.001
High physical activity	36 (38 %)	7 (50 %)	n.s.
Currently smoking	11 (11 %)	2 (14 %)	n.s.
Fasting plasma glucose (mmol/L)	4.8 ± 0.4	4.9 ± 0.5	n.s.
Fasting plasma insulin (pmol/L)	45.7 ± 28.9	44.7 ± 13.4	n.s.
OGTT 2 h plasma glucose (mmol/L)	5.5 ± 1.1	6.4 ± 0.9	0.007
HbA1c (mmol/mol)	32.4 ± 2.2	33.6 ± 2.8	0.067
M-value (GIR/lbm/min 30 min)	13.5 ± 3.8	11.7 ± 3.3	n.s.
M/I (GIR/lbm/min/ pmol/L)	0.02 ± 0.01	0.02 ± 0.01	n.s.
IVGTT AIR (pmol/L x min)	3295 ± 2060	3483 ± 2500	n.s.
IVGTT LIR (pmol/L x min)	7354 ± 4089	8999 ± 4868	n.s.
HOMA-IR (mmol x mU/L^2^)	9.6 ± 6.5	9.6 ± 3.4	n.s.
Serum cholesterol (mmol/l)	4.8 ± 0.9	5.2 ± 0.9	n.s.
Serum HDL (mmol/L)	1.6 ± 0.4	1.7 ± 0.4	n.s.
Serum triglycerides (mmol/L)	1.0 ± 0.5	1.0 ± 0.3	n.s.
Serum LDL (mmol/L)	2.7 ± 0.7	3.0 ± 0.8	n.s.
Serum ALT μkat/L)	0.42 ± 0.25	0.41 ± 0.21	n.s.
Serum CRP (mg/L)	1.16 ± 2.27	1.01 ± 1.15	n.s.
Serum creatinine (μmol/L)	87 ± 15	97 ± 24	0.046
Adipocyte cell size (μm)	92.2 ± 12.4	98.2 ± 16.0	n.s.
Serum adiponectin (μg/mL)	9.2 ± 4.7	8.8 ± 4.0	n.s
Il-6 (ng/mL)	43.4 ± 22.6	51.4 ± 17.5	n.s.

NGT = normal glucose tolerance. IGT = impaired glucose tolerance. IL-6 = interleukin-6. Data are mean ± standard deviation. *P-*values have been calculated as stated in Methods. *P-*values below 0.1 are given numerically, otherwise stated as not significant (n.s.)

**Table 4:** Change in clinical and metabolic parameters, presented in all subjects as well as the subgroup that were normoglycaemic at baseline, stratified according to follow up glycemic tolerance status

	All	NGT-NGT	NGT-IGT/T2D	*P-*values
*N*	135	100	14	
Delta BMI (kg/m^2^)	0.9 ± 2.0	0.7 ± 1.6	1,7 ± 2,3	0,0569
Delta weight (kg)	2.6 ± 6.3	1.9 ± 4.9	4.8 ± 7.1	0.062
Delta fat per cent (%)	0.6 ± 4.2	0.3 ± 4.1	3.4 ± 5.2	0.0203*
Delta fasting plasma glucose (mmol/L)	0.2 ± 1.1	0.1 ± 0.4	0.3 ± 0.6	n.s.
Delta OGTT 2 h plasma glucose (mmol/L)	0.5 ± 2.5	0.1 ± 1.1	2.8 ± 1.3	<0.0001***
Delta HbA1c (mmol/mol)	1.4 ± 6.8	0.5 ± 2.1	1.1 ± 1.7	n.s.
Delta serum triglycerides (mmol/L)	−0.1 ± 0.6	0.0 ± 0.7	−0.1 ± 0.4	n.s.
Delta serum cholesterol (mmol/L)	0.2 ± 0.8	0.2 ± 0.9	0.3 ± 1.1	n.s.
Delta serum HDL (mmol/L)	0.03 ± 0.3	0.05 ± 0.2	−0.1 ± 0.3	0.0858
Delta serum LDL (mmol/L)	0.2 ± 0.6	0.3 ± 0.6	0.4 ± 0.9	n.s.

NGT = normal glucose tolerance. IGT = impaired glucose tolerance. T2D = type 2 diabetes mellitus. Data are means ± standard deviation. Delta values have been calculated subtracting the baseline value from the follow-up value of the same variable. *P-*values below 0.1 are given numerically, otherwise stated as not significant (n.s.)

### Results for the group exhibiting IGT or T2D at follow-up

Independently of baseline glucose tolerance status, 23 individuals exhibited IGT or T2D at the end of follow-up (5.4 and 6.3 years, respectively, n.s.). Their characteristics at baseline, compared to the group that at follow-up were normoglycemic, are given in Table 5. There were significant differences in family history of T2D, fasting plasma glucose levels, OGTT plasma glucose at 120 min, HbA1c and M-value, as well as total cholesterol. As expected, there were significantly larger changes in weight, BMI, fasting plasma glucose, OGTT plasma glucose at 120 min and HbA1c in individuals developing IGT or T2D during the follow-up period (data not shown) than the subjects remaining NGT.

**Table 5:** Clinical characteristics at baseline of the subjects with NGT or IGT/T2DM at follow-up, independent of baseline glycemic tolerance status

	NGT	IGT/T2D	*P-*value
*N*	112	23	
Follow-up time (years)	5.4 ± 2.4	6.3 ± 2.4	n.s.
Age (years)	40.0 ± 6.7	42.6 ± 5.6	0.082
Sex (% male)	51 (46 %)	8 (35 %)	n.s.
Weight (kg)	77.6 ± 13.3	75.0 ± 12.5	n.s.
BMI (kg/m^2^)	25.1 ± 3.4	25.5 ± 3.6	n.s.
Waist (cm)	88.6 ± 10.4	89.7 ± 10.9	n.s.
Waist/hip circumference ratio	0.86 ± 0.09	0.88 ± 0.09	n.s.
Systolic blood pressure (mmHg)	118 ± 11	120 ± 12	n.s.
Diastolic blood pressure (mmHg)	75 ± 9	75 ± 8	n.s.
Fat percent (%)	25.2 ± 8.1	28.0 ± 6.2	n.s.
High heredity for T2D	61 (55 %)	15 (63 %)	0.012
High physical activity	36 (34 %)	9 (41 %)	n.s.
Currently smoking	11 (10 %)	2 (9.0 %)	n.s.
Fasting plasma glucose (mmol/L)	4.8 ± 0.4	5.0 ± 0.7	0.029
Fasting plasma insulin (pmol/L)	48.4 ± 31.2	56.0 ± 36.3	n.s.
OGTT 2 h plasma glucose (mmol/L)	5.8 ± 1.4	7.4 ± 1.5	<0.0001
HbA1c (mmol/mol)	32.2 ± 2.2	33.5 ± 3.2	0.029
M-value (GIR/lbm/min 30 min)	13.3 ± 3.8	10.9 ± 3.7	0.010
M/I (GIR/lbm/min/pmol/L)	0.02 ± 0.01	0.02 ± 0.01	n.s.
IVGTT AIR (pmol/L x min)	3307 ± 1998	2803 ± 2153	n.s.
IVGTT LIR (pmol/L x min)	7552 ± 4085	9011 ± 5482	n.s.
HOMA-IR (mmol x mU/L^2^)	10.3 ± 7.2	12.8 ± 9.3	n.s.
Serum cholesterol (mmol/l)	4.8 ± 0.9	5.3 ± 0.8	0.030
Serum HDL (mmol/L)	1.6 ± 0.4	1.6 ± 0.4	n.s.
Serum triglycerides (mmol/L)	1.0 ± 0.5	1.1 ± 0.4	n.s.
Serum LDL (mmol/L)	2.8 ± 0.8	3.1 ± 0.8	0.079
Serum ALT (μkat/L)	0.43 ± 0.25	0.46 ± 0.35	n.s.
Serum CRP (mg/L)	1.21 ± 2.21	1.08 ± 1.18	n.s.
Serum creatinine (μmol/L)	87 ± 15	93 ± 22	n.s.
Adipocyte cell size (μm)	93.3 ± 12.5	98.0 ± 13.2	n.s.
Serum adiponectin μg/mL)	9.1 ± 4.6	8,4 ± 3,9	n.s.
Il-6 (ng/mL)	43.5 ± 21.7	55.8 ± 19.2	0.085

NGT = normal glucose tolerance. IGT = impaired glucose tolerance. T2D = type 2 diabetes mellitus. IL-6 = interleukin-6. Data are means ± standard deviation. *P-*values below 0.1 are given numerically, otherwise stated as not significant (n.s.)

In this group, there were significant increases in weight, BMI, OGTT plasma glucose levels at 120 min and acute insulin response (IVGTT AIR), while insulin sensitivity (M) significantly decreased. The increase in late insulin response (IVGTT LIR) was not statistically significant (*p =* 0.073). The mean weight increased by 5.8 ± 8.5 kg (*p =* 0.003), mean BMI by 1.5 ± 2.0 kg/m^2^ (*p =* 0.001), mean OGTT plasma glucose at 120 min by 5.9 ± 4.6 mmol/L (*p =* 0.001), mean acute insulin response by 691 ± 645 pmol/L x min (*p =* 0.047), and mean M-value decreased by −1.5 ± 2.2 GIR/lbm/min (*p =* 0.016).

### Predictors

Statistically significant crude (unadjusted) predictors of deteriorating glucose tolerance status were age, family history of diabetes (FH+), family history of hypertension, OGTT plasma glucose levels at 60 min, 90 min, and 120 min, serum creatinine, serum ALP and serum bilirubin (all *p-*values < 0.05). The three strongest predictors were family history of diabetes (*p =* 0.0004) and OGTT plasma glucose level at 90 min (*p =* 0.0040) and at 120 min (*p =* 0.0070).

We also used a logistic regression model to evaluate independent predictors of the development of IGT or T2D from normoglycaemia at baseline (Tables 6 and 7). In Table 6, including 118 individuals’ data, male sex (*p =* 0.03) and low M-value (*p =* 0.01) both reached statistical significance as independent predictors. In Table 7, including more independent variables but only 79 individuals, only high physical exercise (*p =* 0.02) reached statistical significance.

**Table 6:** Logistic regression to predict the probability of developing IGT or T2D. Additional adjustments were done and are presented in Table 7

**Table 7:** Logistic regression to predict the probability of developing IGT or T2D

	

### Correlations with measures of insulin secretion

We also examined the correlation between measures of insulin secretion and adipocyte cell size and serum adiponectin levels. At baseline, there were weak but statistically significant (unadjusted) positive correlations between adipocyte cell size and IVGTT AIR (R^2^ = 0.07) and IVGTT LIR (R^2^ = 0.18), and negative correlations between serum adiponectin concentrations and IVGTT AIR (R^2^ = 0.04) and IVGTT LIR (R^2^ = 0.09), (*p-*values <0.05), when examining the full cohort. When the groups were analyzed separately, only larger adipose tissue cell size was correlated to IVGTT LIR at baseline (*p-*value <0.05) in the group that remained NGT at follow-up.

## Discussion

The results of this prospective cohort study support the concept that the development of T2D in high-risk individuals is indeed multifactorial and that the involved pathophysiological mechanisms are closely linked. Subjects with first-degree relatives with T2D thus have unfavorable body composition as well as reduced insulin sensitivity, beta cell dysfunction, dyslipidemia and, at the trend level, exhibit markers of adipose tissue cell hypertrophy and dysfunction prior to developing IGT/T2D.

Previous cross-sectional studies have demonstrated impairments in glucose metabolism in non-diabetic FDR compared with control groups. Results from the RISC study indicated insulin resistance and beta cell dysfunction in response to an oral glucose challenge, and suggested that beta cell dysfunction is the major defect determining diabetes development in diabetic offspring [3]. The Botnia study concluded that subjects with a family history of T2D displayed lower disposition indices and lower physical fitness, independent of level of physical activity, as well as an impaired capacity of beta cells to compensate for an increase in insulin resistance imposed by an increase in BMI [24]. Results from the EUGENE2 study have also suggested associations between specific genes contributing to dysfunctional beta cells or insulin resistance [8, 9].

Prospectively, it was recently shown in the control group of the ACT NOW trial, that HbA1c and markers of beta cell dysfunction (insulin secretion/insulin resistance index after an OGTT) predicted the development of T2D in patients with IGT during 2.4 years of follow-up [7]. In a Danish population-based study of patients with IGT or IFG the results of fasting laboratory measures and an OGTT showed that hypertension, higher BMI, serum triglycerides and plasma glucose levels predicted T2D during a 3.5 year follow-up period [25]. In a similar study based on OGTT and four years of follow-up, Moromoto et al. proposed that disturbances in insulin secretion had a greater impact on the incidence of type 2 diabetes than insulin resistance in a Japanese population [26]. In the current prospective study with the primary aim to evaluate pathophysiological mechanisms in FDR, we confirm and extend the previous results using state of the art methodology (intravenous glucose tolerance tests and euglycemic hyperinsulinemic clamp) for more exact determination of insulin secretion and insulin sensitivity [27].

Several studies have proposed that adipose tissue dysfunction may contribute to insulin resistance. Key characteristics for a dysfunctional adipose tissue are cellular hypertrophy, impaired adipocyte differentiation and a pro-inflammatory adipokine secretion pattern in addition to remodeling and tissue fibrosis [28]. Recent publications by our group have confirmed that this association is found also among FDR to T2D patients. Yang et al. concluded that adipocyte cell size, a well-known predictor of later development of T2D [29], in addition to BMI, is associated with reduced insulin sensitivity in FDR [14]. Furthermore, in a later publication we could show that healthy and normal glucose tolerant FDR had increased HOMA-IR, adipocyte hypertrophy, adipose tissue inflammation and slightly reduced serum adiponectin levels compared to healthy controls in spite of no difference in BMI or percent body fat [15].

In the present study we show at the trend level that markers of adipocyte dysfunction such as adipocyte cell size and circulating IL-6 are further altered in FDR with manifest IGT (however, not reaching statistical significance), strengthening the concept of adipose tissue dysfunction as a contributor to the development of insulin resistance and T2D. Interestingly, in a detailed study of obese individuals with or without insulin resistance Kloting et al. demonstrated that insulin sensitive obesity was characterized by smaller adipocytes, higher secretion of adiponectin and reduced adipose tissue inflammation, in fact, the strongest predictor of insulin sensitivity was the combination of adiponectin and cellular markers of inflammation, markers that were distinctive also in the present study [30]. Importantly, Andersson et al. recently concluded that reversing adipose tissue dysfunction is possible by weight loss and that this correlates with reduction of the metabolic risk profile. Furthermore, the reduction in subcutaneous adipocyte volume associate more strongly with improvement of insulin sensitivity compared to fat mass reduction *per se* [31].

In our study serum adiponectin levels did not significantly differ between the group of FDR developing IGT/T2DM and the group of FDR remaining NGT. However, Onat et al. showed that serum adiponectin levels did in fact not diminish linearly with increasing BMI [32]. The authors concluded that high serum adiponectin in certain populations fail to exhibit anti-inflammatory properties, and that gender, partially explained by sex hormone binding globulin levels in women, influenced the correlation between serum adiponectin levels and anti-inflammatory markers [32]. This hypothesis could possibly contribute to explaining why serum adiponectin did not reach statistical significance in our study population.

It is important to note, that in this study we have studied subcutaneous adipose tissue biopsies, as opposed to visceral adipose tissue. The latter has been extensively studied and there is consensus today regarding its major role in the development of cardiometabolic disease, including T2D. Large subcutaneous adipose tissue storages have been proposed to be less indicative of insulin resistance and its associated metabolic derangements [33]. However, as shown by Gustavson et al., an inability to store excess energy subcutaneously is associated with the accumulation of visceral fat, and the subcutaneous adipose tissue function thus plays a role in the development of ectopic fat storage [28]. In addition to the conclusion drawn from subcutaneous biopsy data in this study, we found that waist circumference and WHR both tended to be larger, however not reaching statistical significance, in the group that developed IGT/T2D than in the individuals with NGT at follow-up (shown in Table 3 and Table 5), i.e. indicating larger visceral adipose tissue storage.

In this study, we could also show that insulin secretion is correlated with two important markers of adipose tissue dysfunction, adipocyte hypertrophy and reduced circulating adiponectin levels, suggesting a potential cross-talk between adipose tissue and beta-cell function, potentially through endocrine regulation by one or several secreted, that was recently proposed by Cantley et al. [34].

Attention has been brought to the patterns of weight gain prior to the development of T2DM by The Whitehall II Cohort study [35]. The majority of individuals that developed T2DM had only a modest weight gain during the study period, but were overweight during the entire 18 years follow-up. Two other, more extreme weight gain patters were identified and all three groups increased significantly more compared to the control group not developing T2D. FDR are at increased risk of developing overweight or obesity and are, for a given BMI, more likely to display an increased risk profile for both T2D and cardiovascular disease compared to healthy controls without family history of T2D [36]. Higher body fat percentage and waist hip ratio were the strongest predictors for development of IGT and T2D from normoglycaemia at baseline.

Interestingly, high physical activity was a risk factor of IGT/T2D in this cohort, which may seem counterintuitive at first. However, our research group is working on a cross-sectional study on the same cohort of individuals, comparing them to a control group without heredity for T2D, and the preliminary results show that high physical activity is more prevalent in the FDR group than among the controls. This could be due to a selection bias, where the FDR recruited from the general population are aware of their cardiometabolic risk profile, and thus succumb to a physically active lifestyle to minimize the risk of disease. However, Mozaffarian et al. showed a u-shaped relationship between physical and the risk of atrial fibrillation, reminding about the complex associations between lifestyle and cardiometabolic disease [37].

A limitation of this study could be the measurements used to assess dyslipidemia. We evaluated neither apolipoprotein subtypes, nor size of lipoprotein particles, which could have altered the conclusions we reached, i.e., that measures of dyslipidemia did not significantly differ between normoglycemic FDR and FDR developing IGT/T2D. Studies have suggested that, e.g., serum lipoprotein[Lp](a) levels in subjects with an apparently advantageous blood lipid profile, could predict cardiometabolic disease, possibly also mediated by gender differences in autoimmune activation, and thus of interest to investigate in this cohort [38, 39].

Finally, two possible confounders are important to mention. We did not collect data on the individuals’ dietary habits, which could possibly have affected the associations studied here. Another possible confounder is the significantly longer follow-up time in the group that developed IGT/T2D than in the group that remained NGT. The IGT/T2D were thus slightly older than the NGT subjects, and as age is an important risk factor for T2D, the difference in follow-up time could have affected the metabolic differences studied. We also did not stratify our study groups by gender. As gender-specific differences have been demonstrated in several of the parameters measured in this study, this might have affected our results. However, we did account for gender differences when performing the multiple regression analysis.

## Conclusion

In conclusion, individuals with a family history of type 2 diabetes and deteriorating glucose tolerance, showed insulin resistance as well as beta cell and possibly also adipose tissue dysfunction, emphasizing the multifactorial pathophysiology in the development of IGT and T2D.
